# PUBERTY IN A SAMPLE OF BRAZILIAN SCHOOLBOYS: ONSET AND ANTHROPOMETRIC CHARACTERISTICS

**DOI:** 10.1590/1984-0462/2021/39/2019109

**Published:** 2020-08-26

**Authors:** Taciana Carla Maia Feibelmann, Adriana Paula da Silva, Juliana Pereira Pontes Santos, Esthefania Garcia de Almeida, Heloisa Marcelina da Cunha Palhares, Maria de Fátima Borges

**Affiliations:** a Universidade Federal do Triângulo Mineiro, Uberaba, MG, Brazil.

**Keywords:** Puberty, Growth, Boys, Sexual maturation, Puberdade, Crescimento, Meninos, Maturidade sexual

## Abstract

**Objective:**

To determine the age of puberty onset in boys and collect anthropometric data of participants at different puberty stages.

**Methods:**

This is a cross-sectional study that assessed 430 boys in a random sample representing 48,390 students from public and private schools from the city of Uberaba, Southeast Brazil. The inclusion criteria were males, aged between 5 and 18 years, and absence of previous diseases. Participants and their guardians filled a semistructured questionnaire with questions relevant to their and their parents’ puberty. We set the significance at p<0.05 and calculated the 95% confidence intervals.

**Results:**

The mean age found in the puberty stage G2 was 11.2±1.8 (95% of participants in stage G2 were 9.2-13.4 years old). Pubarche data showed a mean of age of 11.0±1.6 years (95% of the participants experienced pubarche when they were 8.0-14.0 years old). When compared to the confidence intervals of two classical studies on the subject, our results showed a trend toward earlier pubarche. In addition, the mean age of this event in the children’s parents was of 12.1±1.4 years, which was significantly higher than the age of the children’s pubarche (p<0.001).

**Conclusions:**

These results indicate a secular decreasing trend in pubarche age and an earlier puberty onset. Considering these parameters, is important to design public policies aimed at preventing these early events.

## RESUMO

**Objetivo:**

Determinar a idade em que a puberdade começa em meninos e coletar dados antropométricos de participantes em diferentes fases da puberdade.

**Métodos:**

Trata-se de um estudo transversal no qual foram avaliados 430 meninos, uma amostra aleatória representativa da população total de 48.390 estudantes de escolas públicas e particulares de Uberaba, Minas Gerais. Os critérios de inclusão foram: ser do sexo masculino, ter idade de 5 a 18 anos e ausência de doenças prévias. Os participantes e seus responsáveis preencheram um questionário semiestruturado com perguntas pertinentes à sua puberdade e à de seus pais. Os dados foram considerados significantes para p<0,05, e os intervalos de confiança calculados foram de 95%.

**Resultados:**

A média de idade encontrada no estágio G2 foi de 11,2±1,8 anos, sendo que 95% dos participantes em G2 tinham entre 9,2 a 13,4 anos. Quanto à pubarca, a média de idade foi de 11,0±1,6 anos, e 95% dos participantes apresentaram pubarca entre 8,0 e 14,0 anos. Quando tais resultados foram comparados aos intervalos de confiança de dois estudos clássicos sobre o tema, houve tendência à pubarca mais precoce. Além disso, a média de idade da pubarca nos pais das crianças foi de 12,1±1,4 anos, significantemente maior em relação à dos filhos (p<0,001).

**Conclusões:**

Os resultados indicam uma tendência secular em direção à diminuição da idade da pubarca e um possível início mais precoce da puberdade. É muito importante considerar esses parâmetros para estabelecer políticas públicas destinadas a prevenir esses eventos iniciais.

## INTRODUCTION

Recent studies have indicated a secular reduction in the age of menarche and thelarche in girls.^1^ ^,^ ^2^ ^,^ ^3^ ^,^ ^4^ ^,^ ^5^ However, it is less clear whether this phenomenon has happened among boys, who usually show the first sign of puberty between nine and 14 years of age.^6^ ^,^ ^7^ Sun et al.,^8^ after analyzing data from the National American Health and Nutrition Examination Survey (NHANES) III, were the first to consider the possibility of a decrease in the age of puberty in boys. Although there has been some criticism, Herman-Guidens confirmed such findings ten years later.^9^ In Brazil, data on the subject are scarce and controversial.^10^ ^,^ ^11^ ^,^ ^12^

The characterization of puberty in boys in epidemiological studies is more complex than in girls, considering it relies on two main methods: one based on pictures, presented by Marshall and Tanner,^7^ and another, more precise, based on measurements that can be taken with an orchidometer.^13^ ^,^ ^14^ ^,^ ^15^ ^,^ ^9^ Therefore, it is difficult to make an accurate comparison between studies.

Despite these barriers, regional investigations addressing these aspects are necessary, given that many environmental factors may be involved in determining the onset and progression of puberty. In addition, the experience of different methodologies to define pubertal stages in epidemiological studies may inspire more research in this field.

Thus, this study aimed to identify the age of puberty onset in a sample including schoolboys from Uberaba, as well as assess if it has an association with the age when such event happened with their parents. We also compared these data with two classic studies: the Harpenden Growth Study and the NHANES III study.^9^ ^,^ ^7^ Considering the importance of ethnicity in puberty onset, we also divided the sample according to ethnicity/skin color.

## METHOD

This is a cross-sectional study that assessed a sample representing 48,390 schoolchildren. The participants were enrolled in public and private schools in the city of Uberaba, Minas Gerais State, Brazil.

Data were collected from February 2012 to September 2013. The Ethics Committee of Universidade Federal do Triângulo Mineiro (UFTM) approved this investigation, under protocol No. 1010. Participants consisted of schoolchildren and their parents or guardians, who signed informed consent forms. The sample size and sampling techniques were calculated in three stages.^16^ ^,^ ^4^ In the first stage, the sample size considered the total population of students. In the second stage, we divided the sample proportionally according to the population distribution among boys enrolled in public and private schools. Finally, in the third stage, we used the cluster sampling technique to determine the number of private and public schools. We randomly selected the schools and schoolchildren to obtain the population sample. The participants received a sealed envelope with the informed consent form after an explanation, and all questionnaires were filled for the study.

The inclusion criteria were males, aged between five and 18 years, and absence of previous diseases. Exclusion criteria were the use of medications or presence of chronic diseases. According to the techniques for sampling calculation, 522 participants would be required to reach the objectives of the study: 85% from public schools and 15% from private schools. However, due to difficulties in obtaining consent from families and children to answer questions related to their sexual maturation, 430 boys were studied, comprising 85% of the proposed sample.

The study participants evaluated their pubertal development, with the help of their parents or guardians when needed. Using the *status quo* method along with the recall method, the schoolchildren answered a semistructured questionnaire, in which they were able to indicate if they had noticed any signs of puberty. If the answer was affirmative, they were asked to inform at which stage they had detected the secondary sexual characteristics. The students also reported at which pubertal stage they were, based on authenticated printed material consisting of photos representing different pubertal stages, as indicated by Marshall and Tanner^7^ and adapted by Chipkevitch.^17^ An orchidometer with rings was used to measure testicles, a method validated by Takihara et al.^18^ The object was disposable and only for individual use. It consisted of five rings with diameters compatible with pubertal stages.^6^

We provided a questionnaire with validated semistructured questions for ethnic stratification. A self-assessment of skin color was used, as suggested by several authors.^19^ Boys were classified as white, black, and non-white/non-black.

If any pathology was observed, the participants were invited to be evaluated at the Endocrinology Ambulatory of UFTM.

Based on the description by Moreno et al.,^20^ the physical examination evaluated height, weight, and six skinfolds.^20^ Body mass index (BMI) was calculated from weight and height, according to the World Health Organization (WHO) tables.^21^ We estimated the body fat percentage (BF%) and used the Deurenberg et al. classification:^22^ extremely low (lower than 6%), low (6.01 to 10%), adequate (10.01 to 20%), moderately high (20.01 to 25%), high (25.01 to 31%), very high (higher than 31.0%). Electronic digital scales (models G-TECH^®^ and BALGL3C; 180 kg capacity and 50 g precision, China) were used to measure weight. A portable Alturaexata^®^ stadiometer (measuring up to 213 cm and with 1 mm precision, Belo Horizonte, Brazil) measured height. Lastly, skinfolds were measured with a CESCORF^®^ scientific skinfold caliper (0.1 mm sensitivity, 85 mm reading range, and ±10 g/mm^2^ pressure, Porto Alegre, Brazil).

General and weighted *Kappa* coefficient correlations^23^ between the physician and participant evaluations were, respectively, 0.631 and 0.817 for pubic hair, 0.40 and 0.533 for genitals, and 0.322 and 0.495 for orchidometer. Although the orchidometer was statistically significant, it had a moderate level of correspondence, which did not allow the use of its data in the analysis and discouraged future research by this method.^23^

Statistical calculations were carried out using Statsoft version 8 and SPSS version 20. We obtained the differences between means using Student’s t-test and analysis of variance (ANOVA), followed by Tukey’s test to compare normal and homogeneous data. We used the Wilcoxon signed-rank test for two dependent samples and the Mann-Whitney test for independent samples to assess nonparametric data. Kruskal-Wallis test calculated more than two means, followed by Dunn’s multiple comparison test. We used the chi-square test to compare categorical variables. When the criteria for using the chi-square test were not entirely fulfilled, Fisher’s exact test was performed instead. Nonparametric variables were correlated using Spearman’s rank correlation coefficient. Next, the variables underwent a descriptive analysis based on absolute and percentage frequencies. For the values calculated in this paper, p<0.05 was statistically significant at a 95% confidence interval (95%CI).

## RESULTS

Data were collected from 430 boys aged between 5 and 18 years. Table 1 describes their characteristics. Regarding secondary sexual characteristics, we evaluated the order of appearance because these results are based on the *status quo* method for gonadarche and the recall method for other characteristics. As the first event, participants presented pubarche at 11.0±1.6 years with 95% aged between 8.0 and 14.0 years; followed by gonadarche at 11.2±1.8 years, 95% aged between 9.2 and 13.4 years; axillary hair at 12.2±1.8 years; facial hair at 13.2±2.1 years, and voice change at 12.6±1.6 years.

**Table 1:** General characteristics of 430 schoolboys from the city of Uberaba evaluated from February 2012 to September 2013.

Characteristics	Groups	Gender
		Male n=430 n (%)
Ethnicity/skin color	White	177 (41.2)
	Black	71 (16.5)
	Not white/not black	182 (42.3)
Social class	Upper	41 (9.6)
	Middle	287 (66.7)
	Lower	102 (23.7)
Nutritional status	Undernourished	14 (3.2)
	Normal	270 (62.6)
	Overweight	64 (14.8)
	Obese	82 (19.0)
% Body fat	Low	13 (3.0)
	Appropriate	245 (56.8)
	High	58 (13.5)
	Very high	114 (26.5)
Pubic hair	P1	162 (37.7)
	P2	81 (18.8)
	P3	40 (9.3)
	P4	64 (14.9)
	P5	83 (19.3)

Table 2 shows the description of the gonadal stage associated with age, height, weight, BMI, and fat percentage at the time of analysis.

**Table 2:** Anthropometric characteristics and Tanner pubertal stage of schoolboys from the city of Uberaba evaluated from February 2012 to September 2013.

Pubertal stage	n (%)	Age (years) mean±SD (95%CI)	Height (cm) mean±SD (95%CI)	Weight (kg) mean±SD (95%CI)	Height (Z score) **mean±SD** **(95%CI)**	BMI (Z score) mean±SD (95%CI)	%BF mean±SD (95%CI)
G1	193 (44.9)	8.8±1.7^a^ (8.5;9.0)	133.4±10.9^e^ (131.8;134.4)	33.0±11.8^h^ (31.3;34.6)	0.4±1.1^l^ (0.2;0.5)	0.6±1.6^m^ (0.4;0.9)	20.2±11.8^n^ (18.5;21.8)
G2	56 (13.0)	11.2±1.8^b^ (10.7;11.7)	146.6±12.1^f^ (143.4;149.9)	42.9±12.9^i^ (39.4;46.3)	0.2±1.1^l^ (-0.1;0.5)	0.6±1.4^m^ (0.2;0.9)	23.7±12.9^no^ (20.2;27.1)
G3	41 (9.5)	13.2±2.0^c^ (12.6;13.8)	156.8±10.3^f^ (153.5;160.2)	53.2±21.12^ij^ (46.6;59.9)	0.1±1.0^l^ (-0.2;0.4)	0.3±1.6^m^ (-0.1;0.8)	28.0±18.0^o^ (22.3;33.7)
G4	97 (22.6)	15.2±1.7^d^ (14.8;15.5)	169.5±9.4^g^ (167.6;171.4)	62.3±16.4^jk^ (59.0;65.6)	0.3±1.0^l^ (0.2;0.5)	0.4±1.4^m^ (0.1;0.7)	22.4±11.5^no^ (20.1;24.7)
G5	43 (10.0)	15.8±1.6^d^ (15.3;16.3)	172.5±7.9^g^ (170.1;174.9)	64.1±11.5^k^ (60.6;67.7)	0.14±1.3^l^ (-0.3;0.5)	0.3±1.2^m^ (-0.1;0.7)	20.0±8.3^no^ (17.4;22.6)
Total	430 (100)	11.7±3.4 (11.3;12.0)	149.4±19.3 (147.6;151.2)	45.9±19.3 (44.1;47.8)	0.3±1.1 (0.2;0.4)	0.5±1.5 (0.4;0.7)	21.9±12.5 (20.7;23.0)

G1-G5: Tanner pubertal stage according to the development of the genitals; n (%): number of subjects and percentage according to the total; cm: centimeters; kg: kilograms; BMI: body mass index; % BF: percentage of body fat; SD: standard deviation; 95%CI: 95% confidence interval. *Different letters indicate a statistical difference between the groups regarding the variables evaluated. † The letters should be interpreted by variable, that is, by column. ‡ a ≠ b ≠ c ≠ d (Kruskal-Wallis test: H {4, n=430}=321.57; p<0.001); e ≠ f ≠ g (Kruskal-Wallis test: H {4, n=430}=308.35; p<0.001); h ≠ i ≠ j ≠ k (Kruskal-Wallis test; p<0.0001); n ≠ o (Kruskal-Wallis test; p=0.004).

The findings above were compared to the mean results and 95%CI found in two classic studies: Harpenden Growth Study conducted by Marshall and Tanner, in 1970,^7^ and Study from the Pediatric Research in Office Settings Network conducted by Hermann-Giddens et al., in 2012.^9^ Table 3 presents the data description and analysis. In this study, the mean age of onset of secondary sexual characteristics was significantly lower than that found in British boys analyzed in Marshall and Tanner’s study.^7^ In addition, age at pubarche was significantly lower than the mean age of Hispanic boys studied by Hermann-Giddens et al.^9^ (Table 3).

**Table 3:** Age of onset of secondary sexual characteristics of schoolboys evaluated from February 2012 to September 2013 in the city of Uberaba and comparison with the results found by Marshal and Tanner^7^ and Herman-Giddens et al.^9^.

Study	Group assessed	n (%)	Evaluated characteristics (years) n (%) mean±SD (95%CI)				
			**G2 stage**	Pubarche	Axillary hair	Facial hair	Voice change
Present study	Brazilian boys	430 (100)	56 (13.0) 11.2±1.8^c^ (10.7;11.7)	268 (62.3) 11.0±1.6^e^ (10.9;11.3)	170 (39.5) 12.2±1.7 (11.9;12.4)	137 (31.7) 13.2±2.1 (12.9;13.6)	159 (37.0) 12.6±1.6 (12.3;12.9)
Marshall and Tanner^7^	British boys	228 (100)	228 (100) 11.6±1.1^c^ (11.5;11.8)	228 (100) 13.4±1.1^g^ (13.3;13.6)	NA	NA	NA
Hermann-Giddens et al.^9^	White American boys	2070 (50.1)	1339 (32.4) 10.1±2.2^b^ (10.0;10.3)	1135 (27.5) 11.5±1.6^f^ (11.3;11.6)	NA	NA	NA
	African American boys	1062 (25.7)	776 (18.8) 9.1±2.1^a^ (8.9;9.4)	694 (16.8) 10.2±1.8^d^ (10.0;10.5)	NA	NA	NA
	Hispanic boys	999 (24.2)	652 (15.8) 10.0±1.8^b^ (9.8;10.3)	540 (13.1) 11.4±1.5^e^ (11.2;11.6)	NA	NA	NA

SD: standard deviation; 95%CI: 95% confidence interval for the mean, n (%): number of subjects and percentage according to the total evaluated by the respective researcher. *Different letters indicate no intercession of confidence intervals between groups in relation to the evaluated variables. †The letters should be interpreted by variable, i.e., by column. NA: not analyzed.

In our sample, we compared three groups of pubertal boys according to their ethnicity/skin color: white (n=109), non-black/non-white (n=94), and black (n=30). We found no difference among the ethnic groups for the studied variables. Table 4 reports the results.

**Table 4:** Age of onset of secondary sexual characteristics according to ethnicity/skin color in schoolboys from the city of Uberaba evaluated from February 2012 to September 2013.

Ethnicity/skin color	n_1_ (%)	Evaluated characteristics (years) n_2_ (%) mean±SD (95%CI)				
		G2 stage	Pubarche	Axillary hair	Facial hair	Voice change
White	177 (41.2)	22 (12.4) 11.5±1.0 (11.1;11.9)	109 (61.6) 11.2±1.7 (10.8;11.5)	86 (48.6) 12.4±1.7 (12.0;12.7)	69 (39.0) 13.3±2.0 (12.8;13.7)	80 (45.2) 12.7±1.7^d^ (12.3;13.1)
Black	71 (16.5)	11 (15.5) 12.1±0.8 (11.5;12.7)	30 (42.2) 11.33±1.58 (10.7;11.9)	23 (32.3) 12.04±1.77 (11.3;12.8)	17 (23.94) 13.53±2.24 (12.4;14.7)	23 (32.4) 12.78±1.83^d^ (12.0;13.6)
Not white/not black	182 (42.3)	23 (12.6) 11.5±1.5 (11.2;12.1)	94 (51.6) 10.9±1.3 (10.6;11.1)	61 (33.5) 12.0±1.5 (11.6;12.4)	51 (28.0) 13.1±2.2 (12.5;13.7)	56 (30.8) 12.4±1.4 (12.0;12.8)

n1: number of subjects according to ethnicity/skin color; n2 (%): number of subjects who presented the evaluated characteristic and percentage according to the total number of subjects mentioned in the line. Kruskal-Wallis test for the G2 stage (p=0.36); pubarche (p=0.15); axillary hair (p=0.39); facial hair (p=0.75); voice change (p=0.17).

We assessed the association between higher prematurity of the onset of secondary sexual characteristics in boys with variables such as BMI. These characteristics showed no differences regarding the age at pubarche and the participants’ age at stage G2 (Spearman’s correlation, r=0.0).

The mean age of students’ pubarche was lower than that of their fathers (11.0±1.6 × 12.1±1.4; Wilcoxon signed-rank test, p<0.001) and mothers (11.0±1.6 × 11.7±1.5; Wilcoxon signed-rank test, p<0.001).

We could not confirm early puberty in seven boys, which would indicate the presence of testicular volume compatible with early puberty by the orchidometer. Among the seven boys who reported early pubarche, such characteristic was confirmed only in two of them, and the exams did not detect pathologies; therefore, it was possibly premature adrenarche.

## DISCUSSION

In this study, 95% of the sample had pubarche at ages between eight and 14 years. Considering that boys usually show their first signs of secondary sexual characteristics at nine years old, our findings suggest that the age of puberty onset in Brazilian boys may be decreasing, similar to girls, as described by Feibelmann et al.^4^ Data have also indicated that the boys studied in the sample had a mean age of pubarche lower than that of their parents. Furthermore, according to data reviewed by Duarte et al.,^12^ compared to research conducted in 1985, in Santo André, São Paulo State, Brazil, our sample presented an earlier age not only of pubarche but also of stage G2 (12.5±1.2 versus 11.0±1.6 and 12.0±1.3 versus 11.2±1.8, respectively).

Similar to our research, after ten years, Gaete et al.^24^ found a trend towards the earlier onset of testicular maturation among Chilean children of medium and low socioeconomic status, but the final age of puberty had no changes. In the same study,^24^ the mean age of stage G2 was 11.2 years, identical to that found in the current investigation. In Karpati et al.,^13^ age at stage G2 was 10.1 years, and pubarche occurred at 11.9 years, which is also similar to the results of this study. In Greek boys assessed by Papadimitriou et al.,^25^ the mean age of G2 was 10.9 years, and pubarche occurred at 11.2 years.

Nevertheless, comparing our study to that of Herman-Guidens et al.,^9^ who identified younger gonadal age among African American and Hispanic boys compared to white boys, the age difference among the ethnic groups was null in our sample. This finding was also contrary to data reported by Sun et al.,^8^ in which non-Hispanic black boys had a lower mean age at onset of sexual maturity when compared to non-Hispanic and Mexican white boys, even though both had maturation in the same age group. The historical miscegenation of the Brazilian population may explain these contrasts. Ethnicity in the studied population is not a determining factor for the onset of pubertal signs.

Several differences among studies on puberty in boys may be partially explained because boys do not have a pubertal event they can measure and remember, such as menarche in girls. In addition, the first manifestation of puberty (i.e., testicular volume increase) may not be observed in most boys, contrary to the thelarche, which is the first sign of puberty in girls and easily identifiable.^6^ These inherent characteristics of male pubertal development make the results found in boys less reproducible among various studies. Different methodologies used for this measure also contribute to this issue, considering that the researcher may use photos or pictures to identify Tanner stages, whether through self-assessment or ectoscopy by the examiner, or perform the physical examination with the support of an orchidometer for testicular measurement.^9^ ^,^ ^7^ ^,^ ^15^

Another controversial issue would be the possible association between overweight and pubertal events in boys. In 2013, Mouritsen et al.^26^ found no association between earlier age of gonadarche and BMI or sum of skinfolds in 90 upper-class boys evaluated in Denmark. On the other hand, Schubert et al.^27^ reported that boys with lower BMI were more likely to present gonadarche before pubarche. A negative association between overweight/obesity (BMI>85^th^ and 95^th^ percentile, respectively) and early sexual maturation was found in NHANES III and National Institute of Child Health and Human Development (NICHD) studies. In contrast, Arquitt et al.^28^ showed that boys with higher BMI concomitantly have higher levels of androgens. However, Wang et al.^29^ revealed that advanced sexual maturation in adolescents was associated with lower subcutaneous fat and BMI. This scenario leads to the question of whether obesity can delay the onset of puberty in boys.

In a recent re-evaluation of the PROS study, the authors found earlier puberty in overweight white and African American boys compared to boys with regular weight. In addition, they identified later puberty in obese boys compared with overweight boys,^30^ although no significant differences were detected for Hispanics boys in Lee et al.^30^ This research found no association between BMI and age at stage G2 or pubarche.

Expected and progressive growth increased during puberty, even though no statistical difference in height was identified between stages G2 and G3 and stages G4 and G5. Also, the Z score for height remained similar from stage G2 to G5. This result demonstrated a good correlation of the sample statistical evolution with current growth curves.^21^

The limitations of this study include its design - a sectional survey (i.e., a sample of students but not the general population) - and use of a self-assessment method to characterize pubertal stages. These factors also limit the comparison with other studies that address this theme.

Nevertheless, some boys had pubarche earlier than expected - before the age of nine. This finding could indicate a secular decreasing trend in pubarche age and an earlier puberty onset. The same data were not found for gonadarche, which could be due to a cross-sectional study limitation. We could not evaluate the onset of testicle growth, only the age of participants at the stage G2.

Data from this study should not be considered for the entire Brazilian population, as the sample was restricted to schoolchildren from Uberaba, a city in a well-developed region in Minas Gerais State. However, the results found and discussed cannot be neglected and indicate the need for longitudinal and multicenter studies. Moreover, the physical examination and testicle volume measurement would be essential to assess puberty in boys in a reproducible and reliable way, as conducted by many researchers from the PROS study.^9^
